# Employing Genome Mining to Unveil a Potential Contribution of Endophytic Bacteria to Antimicrobial Compounds in the *Origanum vulgare* L. Essential Oil

**DOI:** 10.3390/antibiotics12071179

**Published:** 2023-07-12

**Authors:** Francesco Vitali, Arcangela Frascella, Giulia Semenzato, Sara Del Duca, Antonio Palumbo Piccionello, Stefano Mocali, Renato Fani, Giovanni Emiliani

**Affiliations:** 1 Research Centre for Agriculture and Environment, Council for Agricultural Research and Economics (CREA-AA), Via di Lanciola 12/A, 50125 Cascine del Riccio, Italy; francesco.vitali@crea.gov.it (F.V.); sara.delduca@crea.gov.it (S.D.D.); stefano.mocali@crea.gov.it (S.M.); 2 Institute for Sustainable Plant Protection (IPSP), National Research Council (CNR), Via Madonna del Piano 10, 50019 Sesto Fiorentino, Italy; angela.frascella@ipsp.cnr.it; 3 Department of Biology, University of Florence, Via Madonna del Piano 6, 50019 Sesto Fiorentino, Italy; giulia.semenzato@unifi.it; 4 Department of Biological Chemical and Pharmaceutical Sciences and Technologies, University of Palermo, Viale delle Scienze Ed.17, 90128 Palermo, Italy; antonio.palumbopiccionello@unipa.it

**Keywords:** VOCs, endophyte, medicinal plants, biosynthesis pathways

## Abstract

Essential oils (EOs) from medicinal plants have long been used in traditional medicine for their widely known antimicrobial properties and represent a promising reservoir of bioactive compounds against multidrug-resistant pathogens. Endophytes may contribute to the yield and composition of EOs, representing a useful tool for biotechnological applications. In this work, we investigated the genomic basis of this potential contribution. The annotated genomes of four endophytic strains isolated from *Origanum vulgare* L. were used to obtain KEGG ortholog codes, which were used for the annotation of different pathways in KEGG, and to evaluate whether endophytes might harbor the (complete) gene sets for terpene and/or plant hormone biosynthesis. All strains possessed ortholog genes for the mevalonate-independent pathway (MEP/DOXP), allowing for the production of isopentenyl diphosphate (IPP) and dimethylallyl diphosphate (DMAPP) precursors. Ortholog genes for the next steps in terpenoid biosynthesis were scarce. All the strains possess potential plant growth promotion (PGP) ability, as shown by the presence of orthologous genes involved in the biosynthesis of indoleacetic acid. The main contribution of endophytes to the yield and composition of *O. vulgare* EO very likely resides in their PGP activities and in the biosynthesis of precursors of bioactive compounds.

## 1. Introduction

With the increase in the presence of antibiotic-resistant bacteria and the scarcity of new molecules being brought onto the market, alternative strategies are needed to cope with infections from multidrug-resistant (MDR) bacteria [1]. The efficacy of plant-derived bioactive compounds has been investigated to face the challenge of antibiotic resistance [2]. Indeed, as sessile organisms, plants evolved the ability to synthesize natural products, which are viewed as a privileged group of molecules interacting with a wide variety of cellular targets for specific purposes.

Among plant bioactive products, volatile organic compounds (VOCs) composing essential oils (EOs) are receiving much attention in the frame of green chemistry and sustainable practices due to their wide potential applications in different fields, such as agricultural, industrial, and/or medical ones. EOs consist of a composite mixture of fragrant volatile compounds soluble in lipid and organic solvents, referred to as the phytocomplex. The bioactive effects of EOs strongly depend on their composition and the interaction(s) existing among the different compounds [3]. Depending on the plant species, EOs can be synthesized either in all organs, i.e., buds, flowers, leaves, stems, twigs, seeds, fruits, roots, wood, or bark, or in just some of them [4]. Once synthesized, the EO components can be stored in secretory cells, cavities, canals, epidermic cells, and/or glandular trichomes. EO mixtures generally consist of 20–60 different components, mainly terpenes and phenylpropanoids, with two or three of them occurring in high (20–70% of the total content) concentrations [5]. EOs play an important role in plant protection against pathogenic bacteria, viruses, and/or fungi. Moreover, they also prevent insect pest attacks on plants while attracting pollinators and contributing to plant health [4,5,6]. The use of EOs in animals is not associated with harmful side effects; therefore, they have been often proposed as fragrances in the cosmetic industry and also as anticancer, antidiabetic, antioxidant, and anti-inflammatory compounds in medicine [7].

Presently, more than 17,500 species of plants belonging to many angiosperm families, such as *Umbelliferae*, *Lamiaceae*, *Lauraceae*, *Myrtaceae*, and others, are known for their EOs, but only about 300 of them are commercialized [8]. *Lamiaceae* is among the plant families displaying the greatest interest in EO production. It comprises many species rich in flavonoids and terpenes, with diterpenoids being the most abundant. This variety of bioactive compounds grants *Lamiaceae* antioxidant, insecticidal, fungicidal, and bactericidal properties, which confer great potential for economic and pharmacological values [9]. Among the species with aromatic properties, the six best-known vernacular names are thyme, basil, oregano, rosemary, sage, and lemon balm [10]. The antimicrobial activity of EOs obtained from many species of *Lamiaceae* has been investigated, revealing a great effectiveness in inhibiting the growth of different bacteria species, such as *Streptococcus pyogenes*, *Staphylococcus aureus*, *Escherichia coli*, *Listeria innocua*, *Pseudomonas aeruginosa*, *Klebsiella pneumonia*, and *Salmonella enterica*.

The EO obtained from *Origanum vulgare* is known to have multiple beneficial effects and is recognized for its antiseptic, antimicrobial, and antiviral activities [11]. In a previous study, it was shown that the EO distilled from *O. vulgare* L. plants was rich in terpene hydrocarbons [12], in particular the sesquiterpenes germacrene D and β-caryophyllene. Oxygenated terpenes accounted for only 8.6% of the total abundance; among them, only 4-terpineol, caryophyllene oxide, and spathulenol exhibited relative concentrations higher than 1%.

As with all multicellular living systems, medicinal plants can be defined as holobionts in that they host a plethora of microorganisms (especially bacteria and fungi) that can permanently reside in their inner tissues without any visible signs of infection. These microorganisms are referred to as endophytes, and the endomicrobiome of plants is referred to as the phytobiome [13]. Beside the widely reported plant growth-promoting activities, the complex endophytic communities of medicinal plants may contribute directly or indirectly to EO compositions through the synthesis of bioactive molecules, including VOCs, which have the ability to inhibit the growth of other microorganisms (including MDR pathogens) [14].

It has been shown that different *Echinacea* spp. plants grown in the same soil harbor a distinct culturable phytobiome. Moreover, different bacterial species are hosted in all of their compartments (i.e., roots, stem/leaves) as well as in rhizospheric soil [15,16]. In addition to this, it has also been observed that different compartments of the same plant are inhabited by different endophytic bacterial communities and that the degree of strain sharing is extremely low, if not absent [16,17]. Hence, the existence of some forces driving the structuring of such communities [15] was suggested, two of which were later identified as antagonistic interactions and antibiotic resistance profiles [18,19]. Emiliani et al. [17] also proposed the existence of a possible link between the composition of endophytic bacterial communities and the ability of *Lavandula officinalis* EO to counteract the growth of opportunistic human pathogens.

This idea has been confirmed very recently by Polito et al. [20], who showed that some of the compounds identified in *O. vulgare* EOs were also present in the VOC profiles of single bacterial endophytes isolated from different aerial parts of the same plant. In addition, those bacterial endophytes were able to inhibit the growth of opportunistic human pathogenic strains belonging to the *Burkholderia cepacia* complex (Bcc) [20]. Those findings strongly suggested that endophytes may directly contribute (at least in part) to the synthesis of plant EO compounds and to their antimicrobial activity. With these premises, the aim of this work was to evaluate whether the genomic/metabolic repertoire of four bacterial endophytes isolated from different aerial parts of *O. vulgare* [21] could account for the VOCs found in the EO obtained from the same plants (at least in part) [12].

## 2. Results and Discussion

### 2.1. Comparison of VOCs Produced by Endophytic Strains and EO Composition

**Figure 1:**
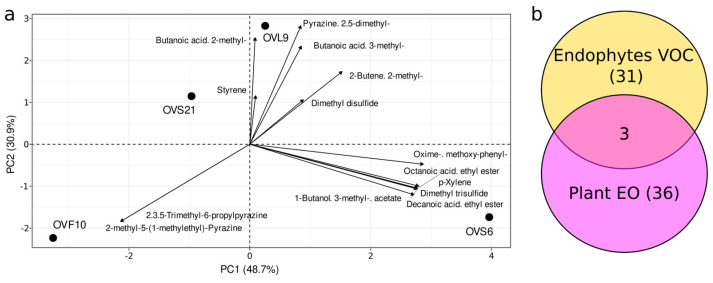
(**a**) Principal component analysis (PCA) of the main compounds in the VOCs of the four endophytic strains. (**b**) VENN diagram reporting the comparison of PubChem annotations of volatile compounds in the *O. vulgare* EO (violet) and in the volatile compounds synthesized by the four endophytic strains (yellow). Numbers represent the number of compounds in the endophytic VOC profiles and plant EO.

The four endophytic bacterial strains used in this work belong to a collection of strains isolated from different aerial parts of *O. vulgare* L. as follows: (i) flower (*Paenibacillus* sp. OVF10); (ii) leaves (*Priestia* sp. OVL9); and (iii) stem (*Metabacillus* sp. OVS6 and *Priestia* sp. OVS21) [12,21]. As previously reported [20], the four strains possess antibacterial activity against different human pathogens, including MDR members of the *B. cepacia* complex. More recently, the VOC profiles of the four isolates grown in pure cultures [20] were obtained. Among the four isolates, OVF10 showed the most specific VOC composition, with the two compounds 2,3,5-trimethyl-6-propylpyrazine (83.92%) and 2-methyl-5-(1-methylethyl)-pyrazine (7.92%) accounting for more than 90% of the total VOC composition. These compounds were not identified in the VOC profiles of the other three strains (OVL9, OVS6, and OVS21), which were more diverse in terms of biosynthesized compounds and their relative contribution to the total VOCs produced. This was confirmed by a principal component analysis (PCA) (Figure 1a) of the compounds, with an average relative abundance higher than 1%. The two strains OVS6 and OVL9, belonging to the same genus (*Priestia*), are located at positive values of the PC1 component, displaying a higher similarity to the other strains even if they produce different compounds. This finding might suggest a correlation between the taxonomical position and VOC profiles of bacterial strains; however, this issue deserves a deeper investigation on a higher number of strains belonging to the same or different genera. As shown in Figure 1a (see position and direction of the arrows, which represent the variable vectors, with respect to the points, which represent VOCs from different strains), six VOCs were associated with OVS6 (oxime-methoxy-phenyl-, octanoic acid ethyl ester, *p*-xilene, dimethyl trisulfide, 1-butanol 3-methyl acetate, and decanoic acid ethyl ester), while the other four were associated with OVL9 (butanoic acid 2-methy-, butanoic acid 3-methyl, pyrazine 2,5-dimethyl, and 2-butene 2-methyl). A total of three PubChem annotated compounds (all of which belong to the family of monoterpenoids) were shared between the endophytes’ VOCs and *O. vulgare* EO (Figure 1b), corresponding to α-pinene (KEGG compounds C06308, C09880, and C06306), *p*-cymene (KEGG compound C06575), and γ-terpinene (KEGG compound C09900).

### 2.2. Exploring the Potential Genomic Contribution of Bacterial Endophytes to VOCs in the EO

A generalized genome mining approach to uncovering the main features (gene modules, antibiotic resistance genes, and hydrocarbon degradation genes) of the genomes of the four strains was recently performed [22]. As the EO is obtained by distillation of the entire aerial part of the plant (stem, flower, and leaves), it is likely that the endophytes from all plant compartments might (differently) contribute to its composition. For this reason, we initially performed the analyses using a comprehensive approach by merging all genes detected in the genomes of the four isolates. In this way, it might also be possible to highlight the eventual existence of a sort of “functional complementation”, i.e., different endophytes might contribute different substrates to the biosynthesis of VOCs.

**Figure 2:**
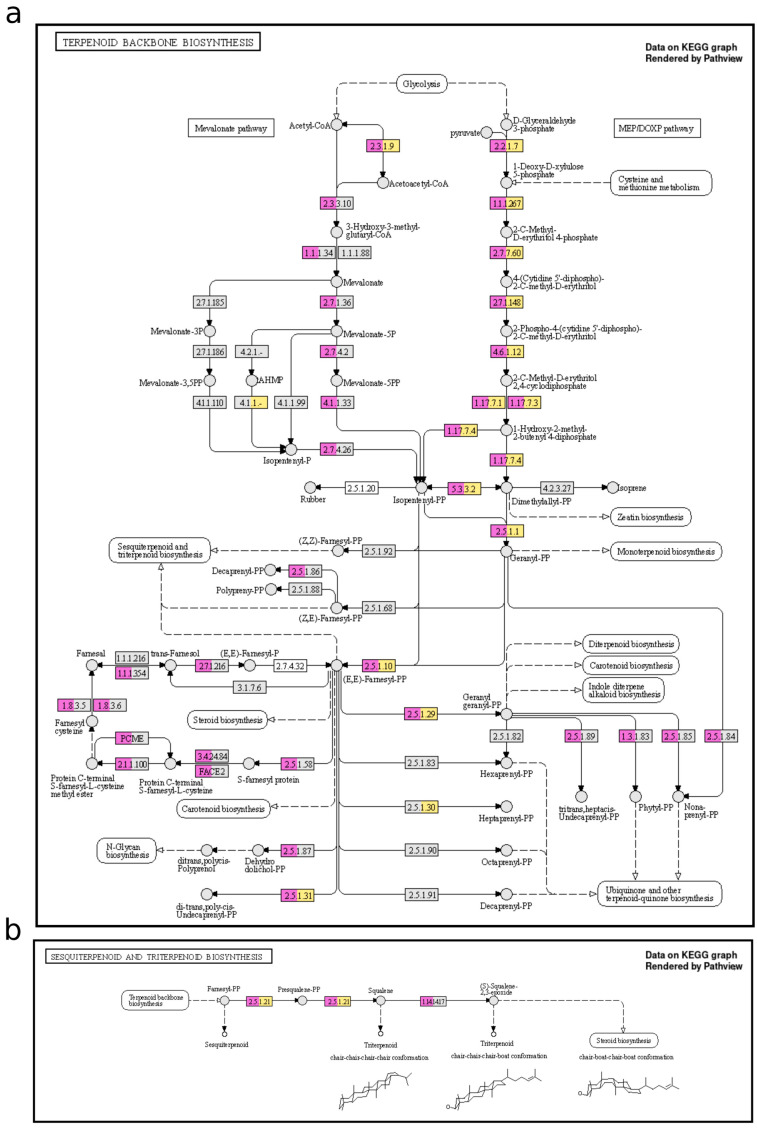
(**a**) Annotated pathway for the biosynthesis of the terpenoid backbone (KEGG map00900), precursor of the other pathways for the biosynthesis of specific terpenoid classes. (**b**) Annotated pathway for the biosynthesis of sesquiterpenoid and triterpenoid (KEGG map00909). In the pathway, genes (rectangular symbols) are annotated based on their presence in the *O. vulgare* genome (violet) or in the list of annotated orthologs from all endophytes (yellow); enzymes in gray are absent either in the *O. vulgare* genome and/or in the list of annotated orthologs of the four endophytes.

**Scheme 1:**
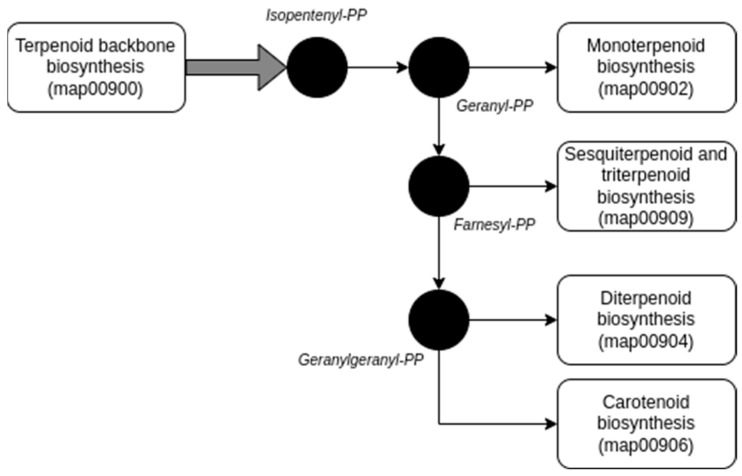
General terpenoid compound biosynthesis scheme.

We first performed the scanning of the endophyte genomes for the presence of genes involved in the biosynthesis of terpenoids, the main constituents of *O. vulgare* EO, which are synthesized by (micro)organisms belonging to all three domains of life through complex biochemical pathways. Their biosynthesis starts with isopentenyl diphosphate (IPP) and its isomer dimethylallyl diphosphate (DMAPP) as the precursors, obtained through either the cytosolic mevalonate (MVA) pathway or the MVA-independent pathway (MEP/DOXP pathway) (Scheme 1 and Figure 2; KEGG map00900) [23]. Both eukaryotes and archaea use the MVA pathway, while bacteria utilize the MEP/DOXP pathway. Moreover, plants carry out the biosynthesis of terpenoids through two pathways: the cytosolic MVA pathway or the plastidial MEP/DOPX pathway. Accordingly, the analysis of the *O. vulgare* genome [24] revealed the presence of the entire gene sets responsible for the biosynthesis of both geranyl-PP and farnesyl-PP (KEGG map00900) (Figure 2a, violet). The analysis of the list of annotated orthologs from the four endophytic strains revealed the presence of the entire gene set involved in the MEP/DOXP pathway (Figure 2a, yellow), coding for enzymes involved in the synthesis of the precursors of (i) zeatin (via DMAPP), (ii) monoterpenoids (via geranyl-PP), (iii) diterpenoids, carotenoids, and indole diterpene alkaloid (via geranyl geranyl-PP), and (iv) sesquiterpenoids and triterpenoids (via farnesyl-PP).

**Figure 3:**
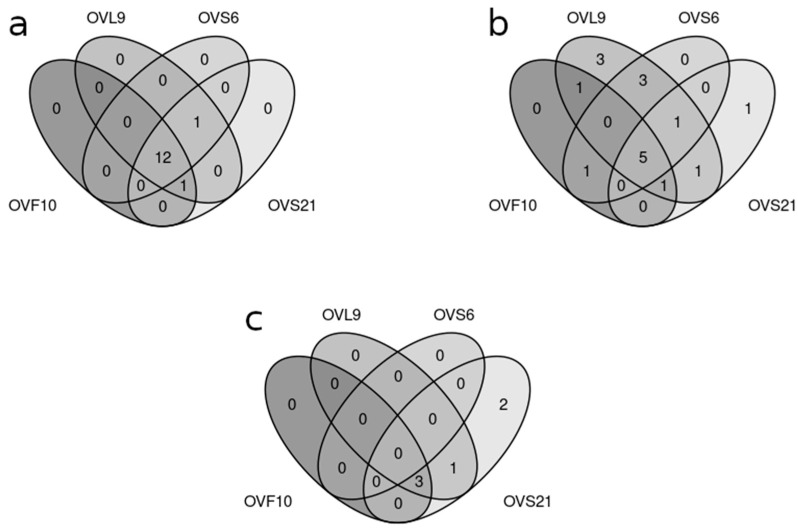
Venn diagrams reporting the number of KEGG orthologs included in the pathway for terpenoid backbone biosynthesis (**a**), tryptophan metabolism (**b**), and carotenoid biosynthesis (**c**) KEGG pathways in comparison between different endophytes.

It has been reported that the composition of *O. vulgare* EO distilled from the same plants from which the four strains were isolated consists of the following four major chemical groups: (i) sesquiterpene hydrocarbons (73.5%), (ii) monoterpene hydrocarbons (17.6%), (iii) oxygenated sesquiterpenes (4.8%), and (iv) 3.7% oxygenated monoterpenes (4.8%) [20]. In addition, the VOC profiles of the four strains grown in pure cultures contained at least three monoterpenoids (α-pinene, *p*-cymene, and γ-terpinene) [20]. Despite this, no genes related to such a pathway were found in the genomes of the four investigated endophytes. However, the synthesis of the numerous monoterpenoid carbon skeletons available in nature is attributed to the wide enzyme class of terpene synthases [24], whose identification and characterization still represent a great challenge in bacteria. Indeed, it has been recently reported that the overall amino acid sequence of such bacterial enzymes does not appear to be similar to those from plants and fungi or to other bacterial terpene synthases [25,26]. Hence, the absence of the monoterpenoid pathway in the four endophytes might only be apparent and attributed to the high diversity existing between plant and bacterial terpene synthases and/or the divergence of annotated bacterial terpene synthase sequences. On the other side, the annotation of sesquiterpenoid and triterpenoid pathways (KEGG map00909; Figure 3b) showed that the endophytes can produce both farnesyl-PP (Figure 3a,b), as a precursor of sesquiterpenoid compounds, and squalene (Figure 3b), as a precursor of the triterpenoid compounds. Moreover, no metabolic potential from the genome mining of the endophytic bacteria was evidenced for the following: (i) the diterpenoid biosynthesis pathway (KEGG map00904), except for a gene coding for ent-kaurenoic acid monooxygenase (EC 1.14.14.107; K04123), which is involved in the biosynthesis of gibberellins; (ii) the carotenoid biosynthesis pathway (KEGG map00906; Supplementary Figure S1), except for the entire module of diapocarotene biosynthesis, which includes genes exclusively available in the endophytic list of annotated orthologs and absent in the host plant.

The data obtained may suggest that the contribution of the four bacterial strains in determining the final *O. vulgare* EO composition might be mainly attributed to the synthesis of precursors for terpenoid biosynthesis since some of them are synthesized by the strains in pure cultures. Indeed, higher plants obtain IPP, a precursor of all terpenoid biosynthesis, using both the cytosolic MVA pathway and the plastidial MEP/DOXP pathway. These two metabolic routes take place in two different compartments of the cell, but they are not completely independent. Indeed, a crosstalk exists between the two biosynthetic routes, consisting of metabolite exchanges among compartments, as experimentally demonstrated using tracer molecules [22]. This suggests that plant cells normally employ molecular machinery and biosynthetic pathways involving plastids in cooperation with their own cytosol. For this reason, the establishment of a system contemplating the presence of bacterial endophytes, which provide precursors to plant cells, is plausible, especially based on the diffusible, volatile, and lipophilic moiety nature of VOCs [27].

#### 2.2.1. Endophytes’ Genomic Basis for Plant Growth Promoting Activity

Apart from the direct biosynthesis of terpenes and/or their precursors, the production of phytohormones and other plant growth promotion (PGP) traits could represent another mechanism through which endophytes can contribute to the EO compositions and yields of aromatic plants, as reported for some auxin-producing bacteria [28]. In this context, Kutlu and colleagues [29] showed that the inoculation of different PGP rhizobacteria in Turkish oregano plants (*Origanum onites* L.) resulted in an increase in plant dry weight with a subsequent increase in total EO yield. Similar results were obtained by Banchio and colleagues [30], who observed an increase in EO yield in Italian oregano (*Origanum* × *majoricum)* inoculated with three PGP rhizobacteria, which was attributed to an enhanced biosynthesis of terpenes. Interestingly, the relative composition of the EO changed significantly as well.

On the basis of these findings, we investigated the presence of endophytes’ genes involved in the production of (i) indoleacetic acid (KEGG map00380, tryptophan metabolism), (ii) abscisic acid (KEGG map0906, carotenoid biosynthesis), and (iii) ethylene (KEGG map00270, cysteine and methionine metabolism). None of the genes required for ethylene and abscisic acid biosynthesis were found in the list of annotated orthologs from all endophytes, while, as expected, they were detected in the plant genome. On the other hand, genes involved in the biosynthesis of indoleacetic acid were detected. This latter result might suggest the PGP potential of the four endophytes, in agreement with previous studies [21]. However, the contribution of such a bacterial phytohormone to the increase in the yield of the endophytes’ host plant EO remains to be assessed.

#### 2.2.2. Strain-Level Differential Genomic Features in VOC Production and PGP Activities

The scanning of pathways reported above was performed on the entire set of genes found in the four isolates to reconstruct the list of annotated orthologs from all endophytes. Nevertheless, it has been reported that one or more selective forces are responsible for the compartmentalization of endophytic bacteria in different anatomical parts of the host plant [16,17,18,19,31]. Therefore, it is also worth making a comparison between the genomes of the individual strains to highlight commonalities or differences.

Regarding the main pathway of terpenoid backbone biosynthesis (KEGG map00900), all four strains possessed all the required genes for completing the biosynthesis of geranyl-PP and (*E*,*E*)-farnesyl-PP (precursors of terpenoid biosynthesis) through the MEP/DOXP pathway. In particular, 12 out of the 63 MEP/DOXP pathway genes were found in the genomes of all the single endophytic strains. This is in agreement with the notion that this pathway appears to be conserved in all prokaryotes [32].

All strains, except for OVF10, also possessed the required genes for the biosynthesis of sesquiterpenoid and triterpenoid (Figure 3a, KEGG map00909), leading to the possible accumulation of presqualene-PP and squalene from farnesyl-PP. In detail, all genomes harbor the gene coding for squalene synthase (KEGG enzyme EC 2.5.1.21), which catalyzes both biosynthetic steps. Among the isolated strains, only OVF10 had a low antibacterial potential toward pathogenic strains belonging to the *B. cepacia* complex, probably due to the low concentration of dimethyl disulfide and dimethyl trisulfide [20]. This strain exhibited a very different VOC profile compared to the other tested endophytes, suggesting that the composition and/or the relative concentration of VOCs might be key players for the anti-Bcc activity. The lower antagonistic activity of OVF10 could also be linked to its inability to fully and efficiently synthesize sesquiterpenoids and triterpenoids, stopping at the biosynthesis of the precursors geranyl-PP and (*E,E*)-farnesyl-PP.

Moreover, all four strains show the genomic potential to synthesize indoleacetic acid, as they possess two ortholog genes involved in the KEGG reactions R02678 (indole-3-acetaldehyde:NAD^+^ oxidoreductase) and R03096 (indole-3-acetamide amidohydrolase), starting from the precursors indole-3-acetaldehyde and indole-3-acetamide, respectively.

Unlike the other examined pathways, the presence of the gene repertoire covering the tryptophan metabolism pathway was dissimilar in the four genomes. Out of the 80 orthologs included in the tryptophan metabolism available in the KEGG pathway database (Figure 3b, map00380), OVF10 possessed the lowest number of orthologs in its genome (8), followed by OVS21 (9), OVS6 (10), and finally OVL9 (15). Only five orthologs were shared by all the isolated strains, while three genes were present in both OVL9 and OVS21. OVL9 was the strain with the most peculiar genomic configuration concerning tryptophan metabolism, with three exclusive genes.

Regarding the biosynthesis of carotenoids (Figure 3c, KEGG ma00906), no orthologs were found in the genome of OVS6, while the highest number of orthologs (six) was detected in the OVS21 genome. Three orthologs were shared between OVS21, OVL9, and OVF10, showing carotenoid biosynthesis potential.

Even if the number of endophytic strains considered in this work does not allow definitive conclusions, the data obtained in this work provide a substantial indication of how, even in the face of endophytic strains with different taxonomies and isolated from different plant compartments, genomic functions are largely in common in relation to the features explored here. The few differences observed between the four endophyte genomes, especially in the case of tryptophan metabolism, might also suggest the existence of metabolic cooperation between endophytic communities residing in different compartments of the same plant.

## 3. Materials and Methods

### 3.1. Genome Sequencing, Assembly, and Annotation

Detailed information on strain isolation and sequencing can be found in the original publications [10,22]. Briefly, endophytes were isolated from different anatomical parts (i.e., flower, leaf, and stem) of *Origanum vulgare* L. in 2018. The genomic DNA was extracted using the PowerLyzer PowerSoil DNA Isolation Kit (MO BIO Laboratories, Inc., Carlsbad, CA, USA) on bacterial cells collected after overnight growth in liquid TSB media. For the genome sequencing, a PCR-free approach with nanopore sequencing was adopted, following a protocol provided by Oxford Nanopore Technologies (ONT) (version NBE_9065_v109_revY_14Aug2019). For the de-novo genome assembly, the Canu software was used, while the genome annotation of the 4 endophytic strains [21] was obtained using Prokka (Galaxy, version 1.14.6; www.usegalaxy.eu, accessed on 27 April 2023) [33]. The complete genome sequences are available in GenBank under the accession numbers CP092335 (OVS6), JALHBO000000000 (OVL9), CP094668 (OVF10), and JALHBP000000000 (OVS21).

In this manuscript, the protein annotations were first used to obtain a KEGG orthologous code (KO) using the online KASS service (https://www.genome.jp/kegg/ko.html, accessed on 27 April 2023) [34]. The obtained KO list for every genome was imported into R software (version 4.2.1) and used to produce annotated KEGG maps using the Pathview package [35]. An annotation analysis throughout the text of this manuscript was performed on the complete list of gene orthologs obtained by joining the 4 separate lists and removing duplicates.

The same procedure was also applied to a publicly available *Origanum vulgare* genome [23], using the annotation file that the authors made available on the DRYAD database [23]. The obtained *O. vulgare* genome annotation was used to produce annotated KEGG maps together with the list of annotated orthologs from all endophytes.

### 3.2. VOC Measurements

Detailed information on the VOC measurements can be found in the original publications [12,21]. Briefly, strains were cultured in TSA media (BioLife, Sarasota, FL, USA) for 48 h at 30 °C, and 2 g of solid medium with colonies was inserted in a headspace vial and sealed. The VOCs were then extracted from the headspace by solid-phase microextraction (SPME) before injection in a gas chromatography (GC) system coupled to an Agilent triple quadrupole mass selective detector (MSD 5973). For each chemical compound, we searched the relative record in the PubChem database (https://pubchem.ncbi.nlm.nih.gov/, accessed on 27 April 2023) [36] to obtain a CAS number. The CAS number was then converted into a KEGG compound number using the CTS—Chemical Translation Service—online tool (https://www.genome.jp/kegg/pathway.html, accessed on 27 April 2023) [37]. The KEGG compound annotations were used to compare the VOCs produced by the different strains with the composition of the EO using Venn diagrams. A principal component analysis (PCA) was also performed to highlight the association between the specific endophytic strains and VOC production.

## 4. Conclusions

Genomic mining approaches coupled with biochemical data and including VOC analyses represent a promising way to study the complex interaction(s) that exist between plant and bacterial endophytes in the production of EO bioactive compounds and to develop strategies and/or models useful for experimental demonstration of the inferred evidence.

The analyses reported in this work represent a further step in the comprehension of the role of bacterial endophytes in medicinal plants’ secondary metabolism and might shed some light on their biotechnological application in pharmaceutical and agricultural fields.

Taking into consideration the list of annotated orthologs from all endophytic strains analyzed in this work, it can be suggested that they may contribute to the yield and composition of *O. vulgare* EO throughout the two following processes: (i) via the biosynthesis of molecule precursors of VOCs detected in the oil, and (ii) by exerting a PGP activity. To investigate the contribution of indoleacetic acid to the increase in the yield of the endophytes’ host plant EO and the production of bioactive molecules, axenic *O. vulgare* plants might be inoculated with bacterial endophytic strains isolated from the same plant. It would then be possible to compare the behavior of inoculated plants with respect to non-inoculated ones from the following different viewpoints: growth, EO yield, production of secondary metabolites, and volatile organic compounds.

This work represents a further step in the comprehension of the complex genomic, phenotypic, and evolutionary processes that are responsible for the endophyte-medicinal plant interaction. In the future, such information might allow to biotechnologically improve this system, i.e., by genetically manipulating bacterial strains or modifying plant–endophyte relationships, to direct the biosynthesis of compounds in EOs. This might be achieved by inoculating synthetic microbial consortia to directly produce bioactive compounds and/or quantitatively stimulate plant growth and qualitatively modulate secondary metabolism.

## Data Availability

Data used for the analysis reported in this manuscript are always freely available as part of the original cited publication.

## Supplementary Materials

The following supporting information can be downloaded at https://www.mdpi.com/article/10.3390/antibiotics12071179/s1, Figure S1: Annotated pathway for the biosynthesis of carotenoid (KEGG map0906).
