# Supplementary figures and images for: Employing Genome Mining to Unveil a Potential Contribution of Endophytic Bacteria to Antimicrobial Compounds in the Origanum vulgare L. Essential Oil
Source: Antibiotics (Basel). 2023 Jul 12;12(7):1179. doi: 10.3390/antibiotics12071179 (PMC10376600; doi:10.3390/antibiotics12071179)

## CAROTENOID BIOSYNTHESIS

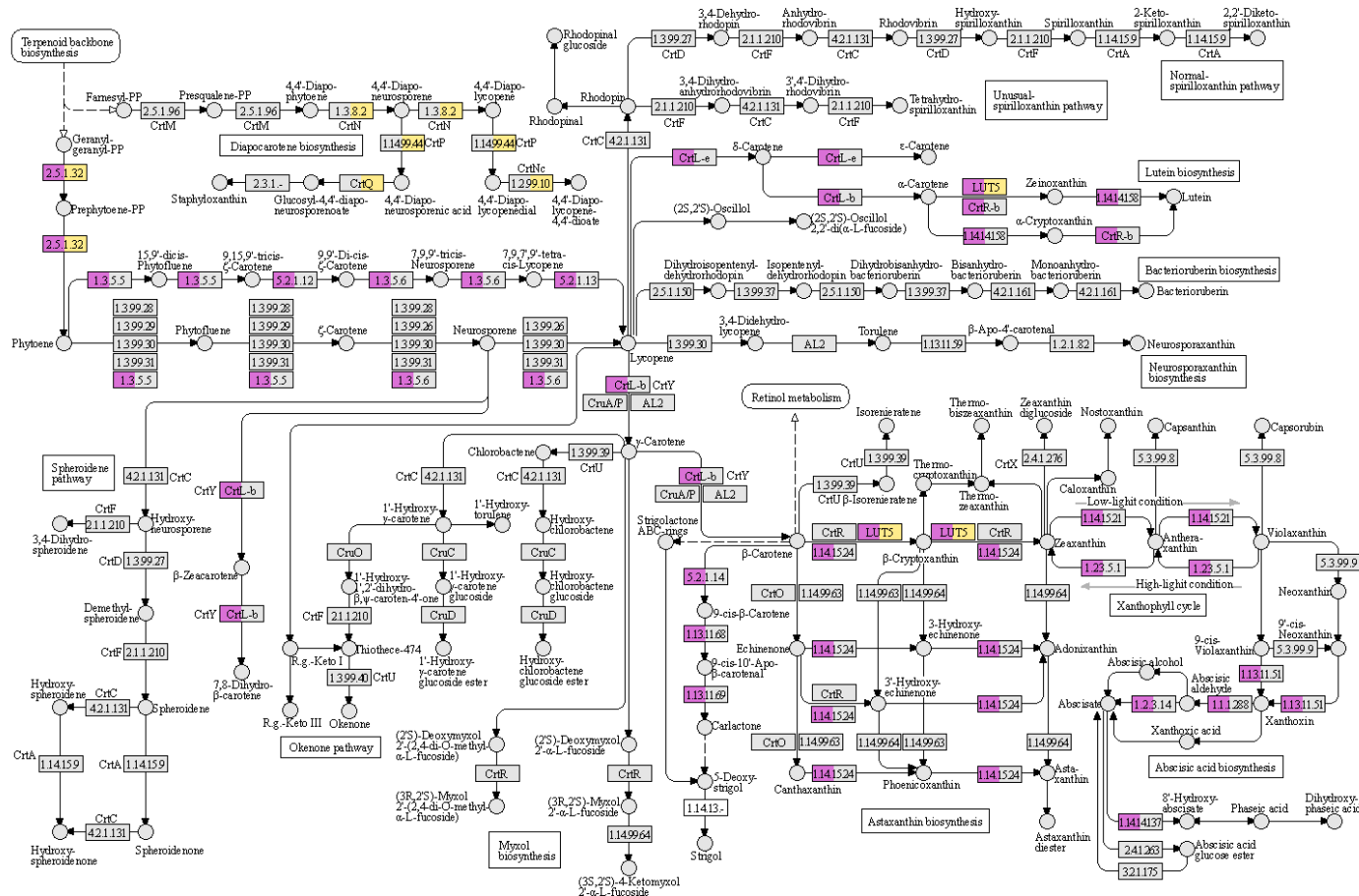

Supplement: Supplementary file 1 [file antibiotics-12-01179-s001.zip › antibiotics-2409984-supplementary.pdf]
